# Supratentorial Gliomas in Eloquent Areas: Which Parameters Can Predict Functional Outcome and Extent of Resection?

**DOI:** 10.1371/journal.pone.0080916

**Published:** 2013-12-05

**Authors:** Giannantonio Spena, Federico D’Agata, Pier Paolo Panciani, Michela Buglione di Monale, Marco Maria Fontanella

**Affiliations:** 1 Neurosurgery Department, Spedali Civili and University of Brescia, Brescia, Italy; 2 Psychology Department and Neuroscience Department of the University of Turin, Turin, Italy; 3 Radiotherapy Department, Spedali Civili of Brescia, Brescia, Italy; The Ohio State University Medical Center, United States of America

## Abstract

**Background:**

To date, few parameters have been found that can aid in patient selection and surgical strategy for eloquent area gliomas.

**Aims:**

The aim of the study was to analyze preoperative and intraoperative factors that can predict functional outcome and extent of resection in eloquent area tumors.

**Patients and Methods:**

A retrospective analysis was conducted on 60 patients harboring supratentorial gliomas in eloquent areas undergoing awake surgery. The analysis considered clinical, neuroradiologic (morphologic), intraoperative, and postoperative factors. End-points were extent of resection (EOR) as well as functional short- and long-term outcome. Postoperatively, MRI objectively established the EOR. χ^2^ analyses were used to evaluate parameters that could be predictive. Multivariate logistic regression analyses were used to evaluate the best combination to predict binary positive outcomes.

**Results:**

In 90% of the cases, subcortical stimulation was positive in the margins of the surgical cavity. Postoperatively, 51% of the patients deteriorated but 90% of the patients regained their preoperative neurological score. Factors negatively affecting EOR were volume, degree of subcortical infiltration, and presence of paresis (P<0.01). Sharp margins and cystic components were more amenable to gross total resection (P<0.01). Contrast enhancement (P<0.02), higher grade (P<0.01), paresis (P<0.01), and residual tumor in the cortex (P<0.02) negatively affected long-term functional outcomes, whereas postoperative deterioration could not be predicted for any factor other than paresis. Subcortical stimulation did not correlate with deterioration, both postoperatively (P<0.08) and at follow-up (P<0.042).

**Conclusions:**

Biological and morphological factors such as type of margins, volume, preoperative neurological status, cystic components, histology and the type of infiltration into the white matter must be considered when planning intraoperative mapping.

## Introduction

Supratentorial gliomas are a heterogeneous group of brain tumors accounting for approximately 30% of all adult primary intracranial tumors and more than half of these are high-grade gliomas (HGGs). These lesions are extremely aggressive, and the vast majority of patients invariably suffer tumor recurrence, with the median survival time ranging from 1 to 3 years after initial diagnosis. Despite facing a better prognosis when compared with higher grade glial tumors, 50–75% of patients harboring low-grade gliomas eventually die of their disease. Median survival times have been reported to range between 5 and 10 years, and estimates of 10-year survival rates range from 5–50% [1]–[3]. For high-grade gliomas, the extent of resection (EOR) is a largely accepted parameter that significantly influence the prognosis both in terms of overall survival and progression free survival [4]–[6]. More recently, robust evidence has supported the importance of gross total resection for the prognosis of low-grade gliomas (LGGs) [7]–[11].

Studies that stress the necessity of achieving a wider larger resection have prompted a discussion regarding the importance of maintaining an adequate postoperative functional status as a goal both in LLGs and HGGs, particularly in “eloquent areas tumors” (EATs). In fact, for HGGs, the short life expectancy and the routine use of adjuvant treatments require a good postoperative performance status because the time in which recovery can occur is short and the treatments can potentially exacerbate deterioration. Some authors [12] have demonstrated that postoperative low performance status can often impede the administration of adjuvant treatments; thus, resulting in decreased survival. Conversely, LGG patients survive longer and are younger; therefore, a permanent deficit will be more difficult to accept because surgery cannot heal this disease.

The increasing use of preoperative and intraoperative brain mapping techniques has radically changed the classical concept of standardized eloquent areas; thus, shifting towards a more individualized approach. For surgical treatment of both HGG and LGG, it is largely recognized that preoperative and intraoperative brain mapping are crucial for maximizing resection while minimizing morbidity [13]–[17]. Unfortunately, it is not clear which pre-, intra-, and postoperative parameters can aid in preoperatively predicting the EOR or the risk of postoperative exacerbation; thus, it is difficult to define patient subgroups. Understandably, the majority of the studies of glioma surgery outcomes had the goal of assessing prognostic factors related primarily to survival and/or tumor progression [18]–[20].

In the current work, we attempted to collect radiological, clinical, and surgical datasets of patients with gliomas in intraoperatively confirmed eloquent areas. The goal was to detect elements that could aid in the prediction of functional outcome and extent of resection in eloquent area tumors.

### Ethics Statement

This retrospective study design was approved by the ethical committee of our Institution (Spedali Civili di Brescia). The need for informed consent from participants was waived by the committee. All patients provided their written and informed consent for all surgical and therapeutic treatments.

### Patients and Methods

This is a retrospective analysis of patients harboring supratentorial gliomas located in presumed eloquent areas, operated on via awake surgery and CSES (cortical and subcortical electrical stimulation) from October 2009 through May 2012. Clinical, surgical, and radiological data collection were obtained via the analysis of inpatient and outpatient charts. Since the study was aimed at further defining the criteria for selecting patients for awake surgery and CSES, only patients who underwent a craniotomy and resection were considered. To increase homogeneity, we did not consider surgical resection of tumors that crossed the midline or had a hemispheric diffusion. Similarly, patients with any of the following characteristics were not considered to be surgical candidates: tumors located in the central region overlapping the rolandic fissure; tumors completely invading pre- or post-central gyri; or tumors invading multiple lobes. In some cases, the patients had undergone a stereotactic tumor biopsy at another institutions before undergoing a craniotomy at our facility. Only patients older than 18 years without any history of previous chemo or radiotherapy were evaluated. Three outcome measurements were calculated: extent of resection (EOR), postoperative neurological status, and neurological status at the six-month follow-up exam.

### Preoperative and Postoperative Clinical and Radiological Data

**Figure 1:**
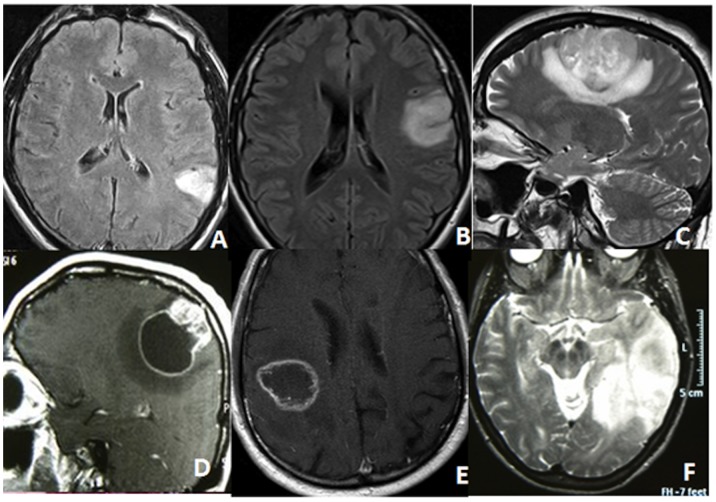
These preoperative MRIs depict the five classes of subcortical infiltration pattern. (A) class 1: tumors invading and confined to only 1 gyrus without infiltration of white matter connections; (B) class 2: tumors invading 1 gyrus with extension to white matter and/or adjacent gyrus; (C) class 3: tumors infiltrating up to 3 gyri and extending toward the long range white matter tracts; (D) the same as class 3 but with a large cystic component; (E) class 4: tumors primarily located in the white matter under eloquent gyri; (F) class 5: lobar tumors.

All patients were evaluated by expert neurologists or neurosurgeons; signs and symptoms were collected and classified as: asymptomatic, seizures, language disturbances, and/or sensory-motor deficits. To express the preoperative functional status and to have a baseline score to be compared to the immediate postoperative and six-month outcome, the Rankin Modified Score (RMS) and KPS were used. A RMS score was assigned during the postoperative period (in the first 30 days post-surgery) and at the 6-month follow-up. Attention was paid to the eventual use of steroids preoperatively and to the effect of the therapy (improvement of symptoms vs. no improvement). It should be noted that we consider improvement after steroid administration as an eventual proof that there was no direct damage to the eloquent area but only an effect of the edema. All the patients underwent gadolinium enhanced MRI, and the T2 FLAIR sequences were specifically used to clarify the extension of LGG. All the tumors were considered to be in eloquent areas based on their anatomical relationships. This means that for language areas, the tumors infiltrated the cortex and/or the white matter of dominant frontal and temporal opercula, supramarginal gyrus (SMG), angular gyrus (AG), middle and inferior parietal gyri (MPG or IPG). For sensory-motor areas, the tumors were considered eloquent if they either infiltrated the cortex and/or the white matter of pre- and post-central gyri or were located in supplementary motor areas (SMA) or the dorsal and ventral premotor cortex (PMC). Clearly, patients with tumors located in premotor areas in the dominant hemisphere also had their language function tested pre- and intra-operatively. We used fMRI for preoperative planning, primarily to determine the activation pattern, the location of the pre- or post-central gyrus, and the approximate distance to the tumor. If the distance from the activation spot is greater than one gyrus or the subcortical infiltration is minimal, we may opt to not perform awake surgery with CSES. For tumors in language areas, we calculate the lateralization index that, together with neuropsychological testing, provides information concerning the dominant hemisphere. Since all the tumors were located inside or in close contact with strongly presumed eloquent areas and the goal of the study was to detect subgroups of patients at higher risk of functional worsening, we analyzed the different types of local infiltration with special attention to growth towards white matter tracts. The visual anatomical limit on MRI to define the infiltration of subcortical connections was the end of the sulcus (Figure 1). The MRI patterns of invasion of the subcortical white matter were classified into 5 groups: (1) tumors invading and confined to only 1 gyrus; (2) tumors invading 1 gyrus with extension to white matter and/or adjacent gyrus; (3) tumors infiltrating up to 3 gyri and extending toward the long range white matter tracts; (4) tumors primarily located in the white matter below eloquent gyri; and (5) lobar tumors. Although this classification is directly related to the tumor volume, it can add further information regarding the relationship between the tumor and the eloquent gyri white matter. Other morphological data used for the analysis were volume (cm^3^), gadolinium uptake, presence of cystic components, margins morphology (sharp or irregular). Preoperative volumes and residual tumor on postoperative MRI were calculated by manual segmentation of T1 contrast enhancement or T2 hypersignal. Because some patients underwent a preoperative MRI at other Institutions, we uploaded the DICOM datasets on our workstation. For HGGs, the postoperative residual tumor was calculated on the basis of the volume of contrast enhancement. For LGGs, the evaluation of the EOR was performed by calculating the volume of the hyperintense signal on FLAIR sequence (preoperative tumor volume – postoperative residue volume/preoperative tumor volume).

Consequently, the EOR was classified as gross total resection (GTR) if the tumor was resected for ≥95% of volume; subtotal resection (STR) if the tumor was removed by 85–95%; and partial resection (PR) if the tumor resection accounted for <85% [22], [23]. Finally, the location of the residual tumor (cortico-subcortical or only subcortical) was established on the postoperative MRI.

### Intraoperative Protocol

All patients were operated on with an awake-asleep protocol after the scalp block was administered and the Mayfield head clamp was inserted. A detailed description of the mapping technique can be found in our previous study [17]. Briefly, a bipolar fork measuring 6 mm in distance between the electrodes, delivers a non-deleterious, biphasic square-wave current in 4-second trains at 60 Hz. Stimulation began at 1 mA and increases by 0.30 mA until generation of contralateral side movement or a paraesthesia occurred. Every positive site was restimulated to confirm reproducibility of stimuli. When tumors are located in language areas, a neuropsychologist administers different tests (picture identification, reading, counting, and writing) and reports the type of disturbance observed (speech arrest, anartria, anomia, reading errors, or acalculia). The patient is unaware of the timing of stimulation, and the current is delivered just before presentation of the slide. After identification of a language error, the patient rests for a short period; then the spontaneous speech and slide reading are tested, and stimulation starts again. For subcortical tumors, we test language or motor areas throughout the subcortical resection, stopping whenever anomalies appear. Parameters such as current intensity, reproducibility of stimuli, and seizure occurrence are observed and registered. With the current study we first reviewed whether the presumed neuroradiologic eloquence was confirmed during CSES and also if the cortical or subcortical positive sites were just in contact with or infiltrated by the tumor (by correlating these sites with the position of the residual tumor on the postoperative MRI). In order to determine whether detection of eloquent sites was associated “per se” with poor functional outcome and restricted resections, these intraoperative findings were then matched with the EOR and postoperative RMS in the statistical analysis.

The histopathologic criteria were established according to the World Health Organization 2007 diagnostic consensus criteria. Grading of the tumors was reported as WHO II, III, or IV where the grade II gliomas were also defined as a low-grade glioma, grade III as an anaplastic glioma, and grade IV as glioblastoma multiforme (GBM). The WHO grade was considered in the statistical analysis and matched with the EOR and functional outcome.

### Statistical Analyses

Statistical analyses and descriptive statistics were performed using SPSS 19.0 statistical software (SPSS, Inc., Chicago, IL). We created different categorical classes for a number of clinical, radiological, and intraoperative parameters that could be predictive of postoperative outcome. When there were less than 6 cases in a category we unified some classes to attain a sufficient number of cases; when this was not possible, we did not consider the parameter because the evaluation could not be considered to be reliable. The parameters were: (1) sex (male or female); (2) age (>40 or ≤40); (3) location (frontal, central area, or temporo-parietal); (4) lesion volume (≤80 cm^3^ or >80 cm^3^); 5) lesion margin (sharp or diffuse); 6) contrast enhancement (yes or no); 7) cystic (yes or no); (8) MRI pattern of infiltration; (9) symptoms (paresis, seizures, or other); (10) preoperative steroid administration (yes or no); (11) grading (WHO II or III or IV); (12) preoperative RMS (equal to 0 or major of 0); (13) functional infiltrated cortex (yes or no); (14) positive subcortical stimulation. χ^2^ analyses were used to evaluate parameters that could be predictive; the significance threshold was set at P<0.05; for multiple comparison, we also computed what parameter survived Bonferroni correction. We used three binary proxies to evaluate positive/negative outcome: EOR total or subtotal; postoperative RMS (worsened or equal/improved compared to Preoperative RMS); and 6 month follow-up RMS (worsened or equal/improved compared to Preoperative RMS).

We examined the relationships between outcome positive indexes by comparing postoperative and follow-up RMS between the EOR total and subtotal groups with a two sample paired t-test. The multivariate logistic regression analyses were used to evaluate the best combination to predict the binary positive outcomes. Odds ratios with 95% confidence intervals were computed. P<0.05 was considered significant. We tried all the different combinations of significant factors using a minimum of 2 different factors and a maximum of 5 different factors. Thus, we computed 112 different models for EOR (combination of 7 factors in models of 2, 3, 4, and 5 factors), 1 model for postoperative RMS (2 factors) and 56 models for follow-up RMS (combination of 7 factors in models of 2, 3, 4, and 5 factors). We chose the models with the best performance for predicting outcome; when models had similar performance, we chose the more parsimonious.

## Results

Table 1 summarizes demographics, clinical characteristics, and MRI findings. Seizures were the most frequent symptom at presentation, followed by motor impairment. The median tumor volume was 59.6 (average: 78.2; range: 4.8–308.3 cm^3^). The class 3 pattern of extension (infiltration of up to 3 gyri plus extension to long-range WM tracts) was the most common; however, the tumors with a volume <80 cm^3^ were predominant, compared to larger tumors (36 vs. 24). Tumors with sharp margins and those with less well-defined borders were approximately similarly represented (52% vs. 48%, respectively). With the goal of reducing neurological deficits, steroids were used in 19 patients (32%). In 17 patients, steroids reduced signs and symptoms. Half (50%) of the patients had a preoperative RMS of 0 and none with a score of 4 was surgically treated (Table 2). Postoperatively, 51.6% of the patients experienced a deterioration of their neurological status; 9 (15%) of these patients suffered aphasia, 18 (30%) suffered motor impairment, and 4 (6.6%) suffered sensory disturbances and apraxia. Among these, only 6 (10%) did not recover their motor impairment to the preoperative status at the six-month follow-up; all of these patients had HGGs (5 GBMs and 1 AA) and all had a residue of tumor.

**Table 1:** Summary of the Demographic, Clinical, and Neuroradiological Features of the Patients.

Age	48±13 (range 72–19)
Sex M/F	21(35%)/39(65%)
Symptoms	
seizures	30 (50%)
paresis	13 (22%)
dysesthesia	8 (13%)
aphasia	3 (5%)
none	6 (10%)
Handedness	
Right	55 (91.6%)
Left	5 (8.3%)
Hemisphere	
left	32 (53%)
right	28 (47%)
Site	
Post-central	18 (30%)
Pre-central	12 (20%)
Frontal opercular	8 (13.3%)
SMA	6 (10%)
SMG - AG	7 (11.6%)
Middle frontal	3 (5%)
Posterior temporal	4 (6.6%)
Temporal opercular	2 (3.3%)
Median tumor volume cm^3^	59.6
<80 cm^3^	36 (53.3%)
>80 cm^3^	24 (46.7%)
Contrast enhancement	
Yes	31 (52%)
No	29 (48%)
Cystic component	
Yes	8 (13%)
No	52 (87%)
Pattern of extension	
1- Single gyrus	4 (7%)
2- Single gyrus+WM	8 (13%)
3- Up to 3 gyri+long range WM tracts	29 (48%)
4- Exclusively in WM	1 3 (22%)
5- Lobar tumor	6(10%)
Type of margins	
sharp	31 (52%)
diffuse	29 (48%)

SMA: supplementary motor area; SMG–AG: supramarginal gyrus-angular gyrus; WM: white matter.

**Table 2:** Overview of Preoperative and Postoperative Rankin Modified Scores and KPS.

	Preoperative	Postoperative	6 months
RMS	N°		
0	30 (50%)	16 (26.6%)	33 (55%)
1	24 (40%)	25 (41.6%)	19 (31.6%)
2	4 (6.6%)	7 (11.6%)	3 (5%)
3	2 (3.3%)	5 (8.3%)	2 (3.3%)
4	0	7 (11.6%)	3 (5%)
Mean RMS	0.6±0.8	1.4±1.3	0.7±1.1
**KPS**	**Preoperative**	**Postoperative**	**6 months**
100	30 (50%)	16 (26.6%)	33 (55%)
90	24 (40%)	25 (41.6%)	19 (31.6%)
80	4 (6.6%)	7 (11.6%)	3 (5%)
70	0	0	0
60	2 (3.3%)	5 (8.3%)	2 (3.3%)
50	0	3 (5%)	1 (1.6%)
40	0	4 (6.6%)	2 (3.3%)
Mean KPS	93.3±8.7	83.7±18	91.6±14.1
% of worsening		51.6% (31 pts)	10% (6 pts)

### Surgical Findings and EOR

Mortality for the group was 0. There was no need for a postoperative urgent craniotomy for intracranial bleeding. In just one case (1.6%), a wound infection occurred, which resolved after antibiotic administration. No cases were converted to general anesthesia and no patients needed ICU monitoring. Intraoperatively, 6 patients (10%) had focal seizures that did not influence the surgery; they were controlled with cold saline irrigation. Only two of these patients suffered seizures preoperatively and just one continued to experience seizures at the six-month follow-up. Antiepileptic drug prophylaxis was not administered preoperatively to patients who had not experienced seizures. Globally, 86.6% of those who suffered seizures before surgery did not experience seizures at follow-up.

In all patients, the presumed eloquent location of the tumors was intraoperatively confirmed by detecting cortical and/or subcortical responsive sites. Some tumors were separated from functional gyri by a sulcus; however, in 54 (90%) it was possible to elicit subcortical responses in the margins of the surgical cavity, demonstrating the close proximity to the critical area. In 17 (45.9%) of these patients postoperative MRI showed residual tumor exclusively located in the WM. The other residual tumors (20 patients; 54%) had a cortico-subcortical location.

The EOR was total in 23 (38.3%) patients, subtotal in 32 (53.3%) and partial in 5 (8.3%). The mean residual tumor volume in the subtotal resection group was 6.5 cm^3^ (range 1.5–13.3 cm^3^), whereas in the partial resection group, it was 22.8 cm^3^ (range: 7.9–37.2 cm^3^). As mentioned above, the residual tumors were predominantly located in cortico-subcortical areas; they were less frequently located in subcortical areas only. Table 3 shows the postoperative MR findings.

**Table 3:** Extent of Resection as Evaluated on Postoperative MRI with Volumetric Analysis and Location of Residual Tumor.

EOR		
Total	23 (38.3%)	
Subtotal	32 (53.3%)	
Partial	5 (8.3%)	
**Volume of residue**	**mean (cm^3^)**	**range (cm^3^)**
subtotal	6.5	1.5–13.3
partial	22.8	7.9–37.2
Residue site (37 pts)		
Cortico-subcortical	20 (54%)	
subcortical	17 (45.9%)	

Histologically, tumors showed malignant features WHO IV malignant features in 17 patients (28%), whereas low grade gliomas (WHO II) accounted for 18 patients (30%). There were 25 anaplastic tumors (42%).

### Statistical Analysis and Prognostic Factors

#### Extent Of Resection (EOR)

The predictive indices as adverse outcome factors for EOR were tumor volume and the pattern of infiltration on MRI with type 3 and 5 being the worst. As mentioned above, it appears that volume plays a role; however, in looking at mean volumes, it also appears that tumors not exceeding certain volumes can infiltrate subcortical tracts and restrict the amount of resection. Tumors that infiltrated the functioning cortex (residual tumor located more cortically) were also associated with unfavorable EOR. This condition is in direct correlation with the presence of a preoperative motor deficit, which is also a negative factor for EOR. Actually, the presence of preoperative paresis can negatively influence EOR in two ways: it is presumably the consequence of infiltration of delicate areas; and the intraoperative worsening of a preexisting deficit can induce the surgeon to prematurely arrest the resection. Steroids are almost always effective in these more severe cases because they counteract the effects of either compression or distortion; thus, their positive effect cannot be reliably considered as proof of a less severe deficit. Conversely, tumors with cystic components and well-defined margins were easier to remove, and these indices were positive outcome factors (Table 4).

**Table 4:** Outcome Based on Clinical, Demographic, and MRI Variables.

Variable	EOR	?^2^	Post-SurgeryRankin	?^2^	Follow-up Rankin	?^2^
Sex	–	0.38	–	0.55	–	0.35
Age	–	0.33	–	0.41	–	0.63
Localization	–	0.26	–	0.33	–	0.95
Sharp margins	POS	<0.01		0.10	POS	<0.01
Volume	neg	<0.01	–	0.17	–	0.29
C. E.	–	0.20	–	0.09	neg	0.02
Cystic	POS	<0.01	–	0.56	–	0.41
MRI index	>2 neg	<0.01	–	0.55	–	0.82
Symptoms	Paresis neg/Seizure POS	<0.01	Paresis neg/Seizure POS	<0.01	Paresis neg/Seizure POS	<0.01
Steroid	–	0.06	–	0.07	–	0.28
WHO	–	0.12	–	0.21	IV neg	<0.01
Preoperative RMS	neg	0.02	–	0.30	–	0.10
FI- cort	neg	<0.01	–	0.42	neg	0.02
SC-pos-STIM	–	0.06	–	0.08	–	0.42

Outcome POS/NEG = χ^2^ (significant: P<0.05); POS = positive outcome factor, NEG = negative outcome factor.

P in bold: significant P<0.05; Bonferroni corrected for multiple comparison.

FI: infiltrated functional cortex.

#### Postoperative and 6-months Rankin Modified Scale (RMS)

For postoperative RMS, the only factor that survived the Bonferroni test was the presence of paresis as a symptom that predicted neurological deterioration (Table 4). Postoperative RMS appeared to be a less reliable index because it does not differentiate between total and subtotal resections (a well-known positive prognostic factor); furthermore, it does not correlate as well between the 6-month RMS (0.56; p<0.01) and the preoperative RMS (0.73; p<0.01). A possible explanation is that the postoperative RMS is probably also influenced by transitory factors that diminish its prognostic value.

For follow-up Rankin, the malignant nature of the tumor (WHO IV, without a clear margin and with contrast enhancement) was a negative outcome factor (Table 4). Total resection appears to also influence functional recovery because all the deteriorated patients at 6 months underwent subtotal resections. In addition, two independent sample t-tests using the total EOR as grouping variable were significant for follow-up Rankin (Table 5). The most practical explanation is that the presence of residual high grade tumors in an eloquent location could negatively impact clinical improvement either by becoming chronically inflamed or by relapsing. Actually, all but one patient experienced a relapse of their tumors at the six-months follow-up.

**Table 5:** Comparison of Outcome Variables.

Variables	EOR subtotal	EOR total	p
Postoperative RMS	−0.9±1.1	−0.5±1.0	0.23
6-months RMS	−0.2±0.8	0.2±0.4	0.03

P in bold: significant (P<0.05).

The best models for multivariate logistic regression are shown in Table 6. For EOR, we obtained 87% correct predictions using lesion margins, lesion volume, seizures, cystic lesions, MRI index 3 and 5. For Postoperative RMS we obtained 70% correct predictions using lesion size and presence of preoperative paresis. For follow-up Rankin we obtained 95% correct predictions using histology, functional infiltrated cortex, and preoperative paresis.

**Table 6:** Multivariate logistic regression for EOR, Postoperative RMS, and Follow-Up RMS.

EOR					
Variables	B	s.e.	OR	95% CI	p
Sharp margins	−1.95	0.95	0.142	0.022–0.913	0.04
Cystic	−4.71	1.58	0.009	0.000–0.202	<0.01
MRI index 3,4,5	1.89	0.91	6.615	1.108–39.481	0.04
Seizure	−2.70	1.05	0.067	0.009–0.521	<0.01
Volume	1.89	1.03	6.646	0.885–49.923	0.06
Postoperative RMS					
Paresis	1.87	0.76	6.466	1.468–28.483	0.01
Follow-up RMS					
WHO IV	2.147	1.464	8.559	0.486–150.773	0.14
FI-cort	3.027	1.455	20.635	1.192–357.269	0.04
Paresis	3.146	1.501	23.237	1.227–440.239	0.04

B = logistic regression beta; s.e. = standard error; OR = odds ratio; CI = confidence interval.

## Discussion

The focus of the present paper was to determine which factors in EATs could be predictive of the EOR and functional outcome. Traditionally, surgical series have analyzed outcomes based on the anatomical location of the tumor (non-eloquent, near-eloquent, or eloquent), with the eloquent location being an intrinsic negative factor for the EOR and postoperative functional status [20], [22], [24], [25]. Intuitively, the prediction of both the EOR and functional outcome is particularly hard to obtain even with the help of modern neuroradiologic advancements such as fMRI and DTI-ft. As a consequence, the use of intraoperative brain mapping has attracted a growing number of surgeons who operate on patients with EATs because it results in improved outcomes. Recently, Jakola et al. [26] postulated that, because eloquence can modify the surgical strategy towards a less extensive resection, it would be desirable to use all the possible methods to achieve a larger resection.

We found that a relevant factor to be considered when indicating this aggressive surgery appeared to be the grade of malignancy. Actually, when an EAT presents with large contrast enhancement, associated with some motor impairment, surgical resection must be very carefully balanced with the risk of insufficient resection and likely further deterioration due to the progression of the residual tumor. It is known that restricted EOR is associated with shorter PFS and OS and that in EATs this condition can also lead to a poor functional outcome and to an eventual worsening of the global prognosis by reducing the possibility to administer adjuvant therapies [27], [32]. In fact, the strategy to achieve a subtotal resection can be better envisioned in a slow-growing tumor where the plastic potential of the brain allows for a repeat procedure on the patient years later that can obtain a larger resection [28]. In the current series total resection was found to be strongly associated with the maintenance of good neurological performance. Although all the tumors were located in critical areas, as intraoperatively confirmed by a positive subcortical stimulation in 90% of cases, partial resection (PR) accounted for only 8.3% of the cases with the rest being total and subtotal resections. This result is important in supporting the concept that intraoperative mapping also allows more aggressive resections of tumors within eloquent areas. Globally, our functional outcomes were satisfactory, with 90% of patients returning to their baseline RMS. Nevertheless, other recent series of patients operated on through awake surgery and CSES for EATs have reported better definitive functional outcomes [29], [30]. This discrepancy is likely due to the fact that those series were based on LGGs, whereas our series dealt with a higher percentage of HGGs. This hypothesis is also supported by the fact that, as reported in our results, seizures as presenting symptoms showed a positive effect on both the EOR and functional outcome as they typically are associated with LGGs.

Although we are currently adopting increased usage of surgical mapping techniques, concepts and results reported in other larger series differ; thus, their conclusions can be misleading. In a recent paper by De Witt Hamer et al. [31], the authors searched the literature for patients operated on for supratentorial gliomas with and without direct mapping. They found that those patients operated through CSES had fewer late severe neurologic deficits and more extensive resection, despite the fact that their tumors were located more frequently in eloquent locations. Conversely, other authors reported that the EOR significantly decreases with the size of the tumor and/or its location at or near eloquent areas [25], [32]. Moreover, Keles et al. found that patients in whom CSES identified subcortical pathways were more prone to develop permanent or temporary postoperative deficits [14]. Kim et al. [22] reported that the intraoperative detection of eloquent areas is a strong predictor of poor functional outcome; therefore, they stated that negative mapping can assure a safer resection. These latter authors did not use subcortical stimulation; therefore, in those cases where cortical eloquent epicenters were detected, they could have lost the opportunity to verify subcortical functional connectivity and potentially avoid damage. This mechanism could explain why, in their experience, patients who underwent positive cortical stimulation were found to have a higher percentage of deterioration. However, in our opinion, the absence of intraoperative cortical or subcortical responsive sites could mean that some of these tumors were actually not classifiable as EATs.

Interestingly, we did not detect any statistically significant difference in EOR and functional outcomes between locations. We must admit that, in this series, some locations were not represented (i.e., insular tumors and basal ganglia tumors) because of the different technical problems related to complex anatomical features and different outcomes. Similarly, the detection of subcortical functional pathways did not result in a negative prognostic factor “per se”, neither in the postoperative period nor at follow-up. As expected, a large portion of the patients operated through CSES experienced neurological deterioration postoperatively as a function of the manipulation of delicate structures; furthermore, none of the preoperative factors but paresis could predict postoperative deterioration. To confirm this, compared to the predictive power of postoperative worsening alone, paresis was also a stronger predictor of poor 6-months follow-up as well. Interestingly, paresis also affects EOR via the aforementioned hypothesized mechanisms hypo.

As reported in recent series [21]–[26], volume is a predictor of restricted resection; however, we found that some other aspects were also involved. In fact, in attempting to determine the role of tumor morphology and the relationship between tumor and brain, we adopted a practical MRI classification that accounted for the degree of diffusion of the tumor to the subcortical connection. Those tumors extending toward subcortical tracts (namely class 3 and 5) were less amenable to gross total resection and this situation was not significantly related to the volume. In regard to those points, Castellano et al. [33] demonstrated that the presence of infiltrated or displaced fascicles on preoperative DTI-ft was predictive of a lower probability of total resection, especially for tumors with a smaller preoperative volume in which an extensive removal could be foreseen. However, we noted that morpho-structural factors related to the biology of the tumor were also involved in determining the EOR. The tumor-brain interface seemed to influence the resection by creating better dissection’s planes, both for non-enhancing and enhancing tumors. Similarly, Talos et al. [34] found that a large tumor volume was associated with a diffuse tumor margin, and oligodendroglioma or oligoastrocytoma histopathologic types were predictors of incomplete resection. They also stated that tumor involvement of some structures such as the cortico-spinal tract is always predictive of reduced resection. Mandonnet et al. [35], proposed a probabilistic map for the prediction of resectability of LGGs based on the residual tumor present on a postoperative MRI. Interestingly, they found that the areas with the highest probability to have residual tumor were located subcortically. In fact, they attributed this result not only to the wide cortical functional variability but also to the remapping of brain function induced by the tumor [36].

Although the current study is limited by its retrospective design and by a limited number of patients, we hypothesize that, in the future, probabilistic maps of the prediction of EOR will also take into account (in accordance with our results) other clinical and morphological factors related to tumor growth rate and biological behavior.

## Conclusions

Gliomas in eloquent areas are challenging tumors that require extensive knowledge of their natural history, anatomic characteristics, and interactions with brain. Technical skills are mandatory but conceptual implications are essential as well. The surgeon must evaluate all the possible clinical, radiological and surgical peculiarities of each individual single patient in order to compose his own experience in predicting risks and benefits. This work cannot solve all the matters regarding EATs; however, it may add additional information regarding the appropriate selection of patients to obtain the best surgical and functional results. Specifically, we obtained 88% correct predictions for the EOR using margin morphology, tumor volume, symptoms, cystic components, and the degree of infiltration of the white matter. For follow-up Rankin, we obtained 95% correct predictions using histology, infiltrated functional cortex, and preoperative paresis. We propose that in the future, because of neuroradiologic advancements, it will be possible to better predict the EOR and outcome. In addition, we foresee that these parameters could be of interest for empowering the decisional pathway.
